# Ageing adversely affects the migration and function of marginal zone B cells

**DOI:** 10.1111/imm.12737

**Published:** 2017-05-04

**Authors:** Vivian M. Turner, Neil A. Mabbott

**Affiliations:** ^1^ The Roslin Institute and Royal (Dick) School of Veterinary SciencesUniversity of EdinburghMidlothianUK

**Keywords:** ageing, B cells, marginal zone, spleen, T‐independent responses

## Abstract

Marginal zone (MZ) B cells are positioned within the spleen to capture blood‐borne antigen and immune complexes and deliver them to follicular dendritic cells in the B‐cell follicles. We show that within the spleens of aged mice antigen capture by MZ B cells, and their ability to shuttle between the follicle and MZ, were impaired. The ability of aged MZ B cells to migrate towards the MZ chemoattractant sphingosine‐1‐phosphate was increased, suggesting that aged MZ B cells had a greater propensity to be retained within the MZ. An extrinsic impairment in aged B‐cell migration towards the MZ was demonstrated using bone marrow chimeras. The follicular shuttling of MZ B cells derived from either young or aged bone marrow was similarly reduced in aged recipient spleens, showing that ageing effects on splenic stromal cells were responsible for the impaired follicular shuttling of MZ B cells. MZ B cells rapidly mount T‐cell‐independent (TI) antibody‐responses to microbial polysaccharide antigen. In aged mice the ability to produce immunoglobulins in response to the TI type 1 antigen TNP‐LPS was impaired. These ageing‐related changes to the MZ and MZ B cells have implications for the clearance of blood‐borne pathogens. Indeed elderly people have increased susceptibility to *Streptococcus pneumoniae*, a TI antigen, and decreased responses to vaccination. A thorough analysis of the mechanisms that underpin the ageing‐related decline in the status of the MZ and MZ B cells will help the design of novel treatments to improve immunity in the elderly.

**Abbreviations**
CR: complement receptor
FDC: follicular dendritic cell
LPS: lipopolysaccharide
mAb: monoclonal antibody
MARCO: macrophage receptor with collagenous structure
MZ: marginal zone
PE: phycoerythrin
S1P: sphingosine 1‐phosphate
SIGNR1: specific intracellular adhesion molecule‐grabbing non‐integrin receptor 1
TI: T‐independent
TLR: Toll‐like receptor

## Introduction

The splenic marginal zone (MZ) in the mouse surrounds the white pulp and comprises a marginal sinus with a network of sinus lining cells through which blood percolates on its way to the red pulp.^1^ Macrophages and B‐cell populations are specifically associated with this network to enable the continuous surveillance and clearance of blood‐borne pathogens, antigens, toxins and apoptotic cells. MZ B cells are a specialized subset of cells situated on the exterior of the marginal sinus. They express B‐cell receptors specific for microbial polysaccharides, Toll‐like receptors (TLR), complement receptors (CR) and can self‐renew.^1^ These features and their MZ positioning enable them to trap and concentrate blood‐borne antigen on their surfaces. MZ B cells also facilitate the delivery of antigen and antigen‐containing immune complexes to follicular dendritic cells (FDC) in the B‐cell follicles.^2^, ^3^, ^4^ The movement of MZ B cells between the MZ and the B‐cell follicle is regulated by their differing surface expression of the chemokine receptor CXCR5 and the sphingosine‐1‐phosphate (S1P) receptors, S1P_1_ and S1P_3_.^3^ Expression of CXCR5 mediates their migration towards CXCL13 produced by FDC and other stromal cells in the B‐cell follicle. Once within the B‐cell follicle, MZ B cells down‐regulate their expression of CXCR5. S1P, abundant in the bloodstream, attracts the MZ B cells back to the MZ through their expression of S1P_1_ and S1P_3_.

The low activation threshold of MZ B cells^5^ enables them to rapidly mount T‐cell‐independent (TI) antibody responses to the microbial polysaccharide antigens of encapsulated bacteria such as *Streptococcus pneumoniae*. These innate‐like B cells act to provide a rapid first line of defence against blood‐borne pathogens, producing antibody of wide specificity before the induction of T‐cell‐dependent high‐affinity antibody responses.

The microarchitecture of the MZ is grossly disturbed in aged mice^6^, ^7^, ^8^, ^9^ and significantly impedes the delivery of immune complexes to FDC.^6^ However, a detailed chronological analysis of the changes to the microarchitecture of the MZ throughout the life course has not been undertaken. Therefore, in the current study spleens were collected from C57BL/6J mice from 2 to 30 months of age at 3‐monthly intervals and the effects of ageing on the microarchitecture of the splenic MZ were determined. A structurally sound MZ is important for the efficient generation of TI immune responses,^10^, ^11^ and the immature status of the MZ in infants under 2 years old is associated with defective TI antibody responses.^12^ Deficiencies in MZ B cells are also associated with elevated risk of pneumococcal infection and poor antibody responses to capsular polysaccharides.^12^, ^13^ Invasive pneumococcal disease from *S. pneumoniae* infection is a leading cause of mortality in people > 65 years old,^14^ and the efficacy of vaccines against this disease is decreased in the elderly.^15^ Although many studies have addressed the ageing‐related changes to the thymus, T cells and T‐cell‐dependent antibody responses, nothing was known of how ageing influenced the function of MZ B cells and their rapid induction of TI antibody responses. Therefore, in the current study, experiments were designed to thoroughly determine the effects of ageing on the migration and function of MZ B cells.

## Materials and methods

### Mice

Female C57BL/6J mice were purchased from Charles River (Margate, UK). Mice were maintained in‐house under specific pathogen‐free conditions. All experimental procedures were approved by The Roslin Institute's Ethical Review Committee, and were conducted under the authority of the UK Home Office Animals (Scientific Procedures) Act 1986.

### Flow cytometry

Splenocytes were made into a single‐cell suspension, red cell lysed and processed on ice during staining. The following antibodies were purchased from BioLegend (San Diego, CA): anti‐CD1d (1B1), anti‐CD3e (145‐2C11), anti‐CD21/35 (7E9), anti‐CD23 (B3B4), anti‐CD45R/B220 (RA3‐6B2), anti‐CD93 (AA4.1), anti‐CD185/CXCR5 (L138D7). The following antibodies were purchased from BD Biosciences (Oxford, UK): anti‐CD16/32 (2.4G2) and anti‐TNP (G235‐1). Anti‐S1P_1_/EDG‐1 (713412) was purchased from R&D Systems (Minneapolis, MN). After immunostaining, cells were analysed using an LSR Fortessa with diva software (BD Biosciences, London, UK). Cells were gated on lymphocytes and doublets were removed, then data were analysed using flowjo (FlowJo, LLC, Ashland, OR).

### Immunofluorescence

Frozen sections 6–8 μm thick were fixed in ice‐cold acetone, rehydrated in PBS and blocked with normal horse serum before antibody application. The following antibodies were purchased from BioLegend: anti‐CD1d (1B1), anti‐CD4 (RM4‐5), anti‐CD21/35 (7E9) and anti‐CD45R/B220 (RA3‐6B2). The following antibodies were purchased from BD Biosciences: anti‐CD35 (8C12), anti‐MAdCAM‐1 (MECA‐367) and anti‐TNP (G235‐1). Anti‐CD169 (MOMA‐1) and anti‐MARCO (ED31) were purchased from Bio‐Rad (Hemel Hempstead, UK). Anti‐CD209b/SIGNR1 (eBio22D1) and phycoerythrin (PE)‐conjugated anti‐Armenian hamster IgG were purchased from eBiosciences (ThermoFischer, Loughborough, UK). Anti‐CXCL13 (polyclonal) was purchased from R&D Systems. Streptavidin Alexa Fluor 594, goat anti‐rat IgG (H+L) Alexa Fluor 594, donkey anti‐goat IgG (H+L) Alexa Fluor 647 and goat anti‐rat IgG (H+L) Alexa Fluor 488 were purchased from ThermoFisher Scientific (Waltham, MA). Dako fluorescent mounting medium (Agilent, Santa Clara, CA) was used to apply coverslips before image acquisition. A Zeiss LSM5 Pascal (Carl Zeiss, Oberkochen, Germany) upright microscope with zen software (Rochdale, UK) was used for image collection.

### Image analysis

Images were analysed using image J software (NIH, Bethesda, MD). Measurements and disruption scorings were performed as described in the Supplementary material (Fig. S1). Typically, from each spleen from each mouse six to eight images were analysed with at least three mice per age group analysed. Full details of all *n* values for each parameter measured are provided in each figure legend. For example, using this process typically > 40 measurements/mouse were collected for the depth of SIGNR1, MARCO, CD1d and CD169, and > 20 measurements/mouse were made for the area of CXCL13.

### CD21‐PE and TNP immunizations

Mice were given 1 μg of anti‐CD21/35‐PE (7G6, BD Biosciences) intravenously for assessment of CD21‐PE uptake. Short‐term trinitrophenyl (TNP) immunization mice were given 100 μg of TNP‐Ficoll (Biosearch Technologies, Novato, CA) intravenously. Long‐term immunization mice were given 50 μg of TNP‐lipopolysaccharide (LPS) (Biosearch Technologies) or 25 μg of TNP‐Ficoll intravenously.

### Bone marrow chimeras

Bone marrow cells (10^7^ cells) from 2‐month‐old or 18‐month‐old mice were transplanted into lethally irradiated (10 Gy) 2‐month‐old or 18‐month‐old recipient mice. Mice were analysed 10 weeks after reconstitution.

### TNP ELISAs

High‐binding 384‐well plates (Grenier Bio‐one, Kremsmünster, Austria) were coated overnight with TNP‐BSA (Biosearch Technologies) at 10 μg/ml. Plates were washed between each step with PBS containing 0·05% Tween. Blocking was performed using 4% BSA. Serum was diluted 1 in 50 in PBS with 1% BSA. The secondary antibodies anti‐IgG3 (R40‐82, BD Biosciences) or anti‐IgM (RMM‐1, BioLegend), were then applied in a solution of 1% skimmed milk and 1% BSA. Streptavidin‐AP (Roche, Basel, Switzerland) was added in 0·1% BSA. Finally *p*‐nitophenyl phosphate (Sigma‐Aldrich, St Louis, MO) at 1 mg/ml in NPP buffer was applied and plates were read at 405 nm using a Biotek plate reader (Biotek, Winooski, VT).

### Chemotaxis assays

Protocol was adapted from previously published procedures^2^, ^16^ as follows. Splenocytes were made into a single‐cell suspension by passing through a 0·7‐μm cell strainer (ThermoFisher Scientific) and red cell lysed. Adhesive cells were removed by incubating cells in RPMI‐1640 with 5% fetal calf serum for 30 min. Non‐adherent cells were collected and rested on ice in migration medium (RPMI‐1640 with 0·1% BSA) for 1 hr. After resting equal numbers of cells from three young or aged mice were pooled and 1 × 10^6^ cells were placed into the upper chamber of 3 μm Transwells in a 24‐well plate (Corning, Corning, NY). The lower chamber contained either 10, 100 or 500 nm S1P (Sigma‐Aldrich), 500 ng/ml, 1 μg/ml or 3 μg/ml CXCL13 (R&D Systems), or media alone. Three technical replicates were performed. Cells were incubated at 37° for 4 hr before the upper chamber was discarded and cells in the lower chamber were collected and stained for flow cytometric analysis. For relative cell counts, cells were resuspended in 200 μl staining buffer and acquired for 45 seconds at medium speed on an LSR Fortessa. Migration was then calculated as a percentage of input cells.

### HPLC

Concentrations of S1P were measured by HPLC‐MS/MS, using a Dionex Ultimate 3000 HPLC system interfaced to a Bruker Amazon ETD ion trap mass spectrometer. Synthetic S1P was obtained from Sigma and was dissolved in methanol, with heating, to 1 mm. This stock solution was diluted into artificial plasma (50 mg/ml BSA in PBS) to give a 3 μm solution, which was serially diluted threefold into artificial plasma thereby yielding calibration standards of 3000, 1000, 333, 111, 37, 12 and 4 nm.

All subsequent procedures were performed in siliconized tubes or glass vials. To 10 μl of either calibrant solution or murine plasma samples were added 55 μl of TBS (50 mm Tris, 0.15 mm sodium chloride) followed by 200 μl of ice‐cold methanol. The mixture was vortexed for 20 seconds, sonicated for 10 seconds then vortexed for a further 20 seconds. The proteinaceous precipitate was removed by centrifugation (5 min, 4°, 16 000 ***g***) and the supernatant was transferred to glass HPLC vials for analysis.

Five‐microlitre aliquots were injected onto an ACE Ultracore 2·5 Super C18 HPLC column (2·1 mm internal diameter, 75 mm length) heated to 40° in a column oven. The mobile phase constituted buffers A: 0·1% (v/v) formic acid in water and buffer B: 0·1% (v/v) formic acid in methanol. The flow was held at 200 μl/min and the column was pre‐equilibrated at 90 : 10 A : B for 5 min. After injection, the chromatographic separation was developed by washing at 10% B for 1 min before ramping to 100% B over 3 min. The buffer was held at 100% B for 4 min then returned to 10% B over 30 seconds for the next analysis. The mass spectrometer was operated in positive‐ion, multiple‐reaction monitoring mode. S1P was quantified using the transition m/z 380·3 to > 264·3 (fragmentation amplitude 1·0, cut off 103, maximum trap accumulation, 200 ms).

### Proliferation of B cells

B cells were enriched from spleens after red cell lysis using the mouse MACS Pan B Cell Isolation Kit (Miltenyi Biotec, Bergisch Gladbach, Germany). Flow cytometry was used to verify that the sample contained > 90% B cells. B cells were labelled with Cell Trace Far Red (Life Technologies, Carlsbad, CA) and 10^5^ cells were placed into 24‐well nunc plates (ThermoFisher Scientific) in either medium alone or with 10 μg/ml LPS (Sigma‐Aldrich) for 48 hr.

### Statistical analysis

Statistical analysis was performed using graphpad prism (GraphPad Software, San Diego, CA). Polynomial curves were chosen via *F*‐test.

## Results

### Effects of ageing on the microarchitecture of the MZ

The splenic MZ undergoes significant histological changes with age. Our laboratory has previously demonstrated this in 20‐month‐old C57BL/Dk and RIII mice,^6^, ^7^ and an independent study has reported disturbances to the MZ in C57BL/6 mice > 12 months old.^9^ Others have also demonstrated changes to the MZ of 17‐ to 18‐month‐old BALB/c mice.^8^ However, a detailed chronological analysis of the changes to microarchitecture of the splenic MZ throughout the life course has not been undertaken. Therefore, spleens were collected from C57BL/6J mice from 2 to 30 months of age at 3‐monthly intervals and immunohistological and morphometric analyses were used to determine the chronological onset of the ageing‐related cellular disturbances to the splenic MZ.

**Figure 1:**
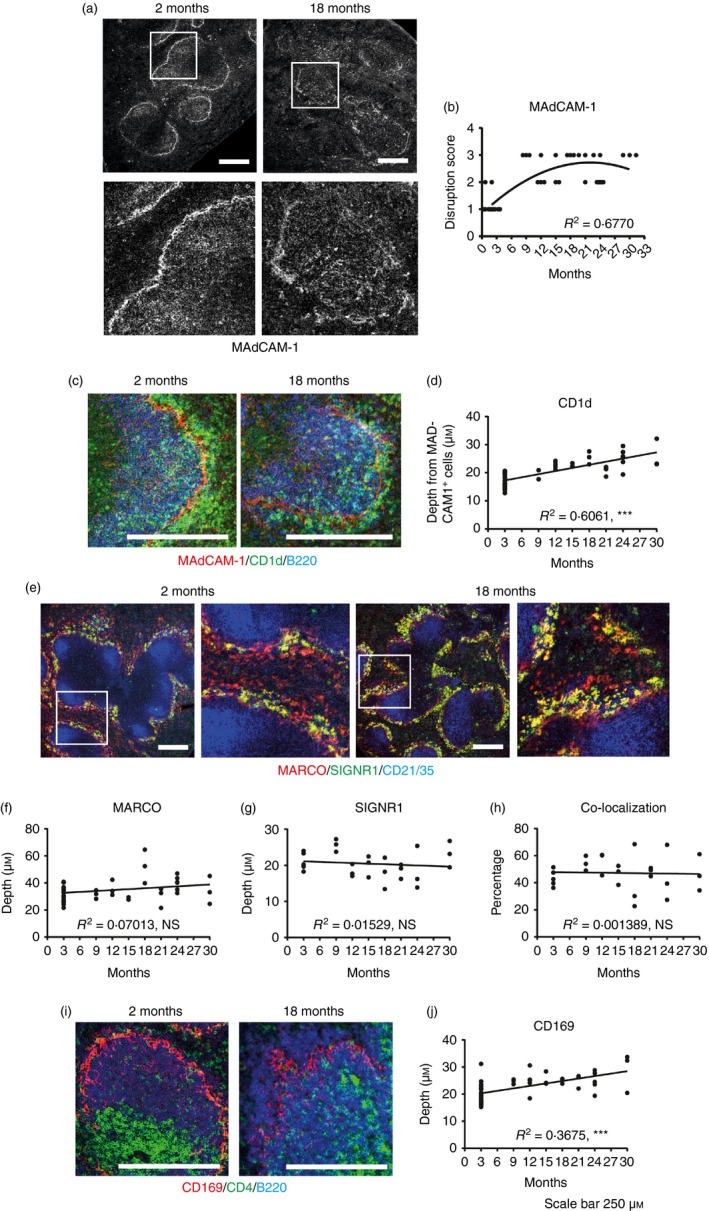
Histological analysis of changes to splenic structure with age. Immunostained spleen sections from mice 2 months to 30 months of age were analysed. (a) Representative staining of MAdCAM‐1 on 2‐month and 18‐month spleens. (b) Quantification of the disruption of MAdCAM‐1^+^ cells at the ages indicated overlaid with a second‐order polynomial. (c) Representative staining of CD1d (green), B220 (blue) and MAdCAM‐1 (red) on 2‐month and 18‐month spleens. (d) Measurement of the depth of CD1d^+^ cells from the MAdCAM‐1^+^ cells at the ages indicated. (e) Representative staining of MARCO (red – marginal zone macrophages), SIGNR‐1 (green – a subset of marginal zone macrophages) and CD21/35 (blue – follicular dendritic cells and B cells) on 2‐month‐old and 18‐month‐old mice. (f) Measurement of the depth of MARCO ^+^ cells at the ages indicated. (g) Measurement of the depth of SIGNR‐1^+^ cells at the ages indicated. (h) Percentage of MARCO ^+^ that are SIGNR‐1^+^ at the ages indicated. (i) Representative staining of CD4 (green – T cells), B220 (blue – B cells) and CD169 (red – marginal metallophilic macrophages) on 2‐month‐old and 18‐month‐old mice. (j) Measurement of the depth of CD169^+^ cells at the ages indicated. *n* = 3 to *n* = 6 except in the 2‐month group where *n* = 23. Except for (b), graphs are overlaid with linear regression curve. *R* ^2^ values shown on bottom right of graphs based on curve fit. ***P < 0·001. Scale bars = 250 μm.

The MAdCAM‐1^+^ MZ sinus lining cells are key stromally derived delineators of the boundary between the MZ and the B‐cell follicle (Fig. 1a). Significant disturbances to the ring of MAdCAM‐1^+^ MZ sinus lining cells were evident from as early as 9 months of age (see Supplementary material, Fig. S1 provides details of disruption scoring), as these cells no longer formed a smooth continuous barrier between the MZ and follicle (Fig. 1a, b). We also assessed the impact of these structural changes on the positioning of distinct leucocyte populations within the MZ. In young mice (2 months old) CD1d^+^ B220^+^ MZ B cells were present within the MZ and B‐cell follicles (Fig. 1c). However, from 12 months of age the depth of the MZ B cells from the MAdCAM‐1^+^ sinus lining cells was significantly increased (Fig. 1c, d).

The MZ contains two distinct populations of macrophages normally situated within separate continuous layers on either side of the MZ sinus lining cells. In young mice the outer layer contains a ring of MZ macrophages that express the scavenger receptor MARCO (macrophage receptor with collagenous structure) and may also express the C‐type lectin SIGNR1 (specific intracellular adhesion molecule‐grabbing non‐integrin receptor 1) (Fig. 1e). Ageing did not significantly alter the distribution of these MARCO^+^ or SIGNR1^+^ MZ macrophages (Fig. 1f, g), nor the percentage of MARCO^+^ macrophages that were also co‐expressing SIGNR1 (Fig. 1h).

The inner layer of the MZ in young mice contains a ring of sialic‐acid‐binding immunoglobulin‐like lectin 1 (SIGLEC1/CD169)‐expressing MZ metallophilic macrophages (Fig. 1i). The distribution of these cells was significantly disrupted in aged spleens as they no longer formed a smooth continuous barrier, and instead displayed a significantly increased depth and decreased density within this region (Fig. 1j).

Overall these data demonstrate that the distribution of MAdCAM‐1^+^ MZ sinus lining cells, MZ metallophilic macrophages and MZ B cells is significantly disturbed in the spleens of aged mice. Although many of these changes were most prominent in mice ≥ 18 months old, significant disruption to the MAdCAM‐1^+^ MZ sinus lining cells was evident from as early as 9 months old and preceded the changes to MZ B cells observed at 12 months. Hence these data show that significant ageing‐related changes are evident in the splenic MZ much earlier than has been previously described.^6^, ^7^, ^8^ The timing of the changes observed also suggests that the ageing‐related disturbances to the distribution of the MAdCAM‐1^+^ MZ sinus lining cells may lead to subsequent disturbances to the distribution of MZ B cells and MZ metallophilic macrophages.

### Effects of ageing on B‐cell populations

**Figure 2:**
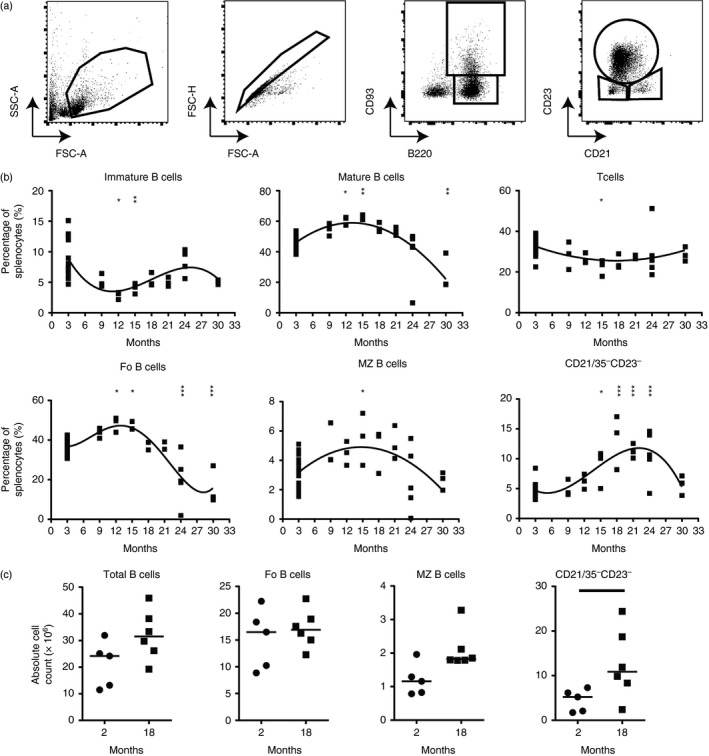
Effect of age on murine B‐cell populations. Flow cytometry was performed on splenocytes from mice aged 2 months to 30 months. (a) Gating strategy to identify B‐cell populations. (b) Percentage of splenocytes that are T cells (CD3e^+^, second‐order polynomial, *R* ^2^ = 0·2515), Immature B cells (B220^+^ CD93^+^, third‐order polynomial, *R* ^2^ = 0·3919), Mature B cells (B220^+^ CD93^−^, second‐order polynomial, *R* ^2^ = 0·5858), follicular B cells (B220^+^ CD93^−^ CD21^mid^ CD23^hi^, fourth‐order polynomial, *R* ^2^ = 0·7130), marginal zone B cells (B220^+^ CD93^−^ CD21^hi^ CD23^lo^, second‐order polynomial, *R* ^2^ = 0·2903) or CD21/35^−^ CD23^−^ B cells (B220^+^ CD93^−^ CD21^lo^ CD23^lo^, third‐order polynomial, *R* ^2^ = 0·6417). *n* = 3 to *n* = 5 except in the 2‐month group where *n* = 23. Results were further analysed via one‐way analysis of variance with Dunnetts post‐test with groups compared to the 2‐month control group for statistical significance. (c) Quantification of absolute total (B220^+^), follicular (B220^+^ CD21^mid^ CD23^hi^), marginal zone (B220^+^ CD21^hi^ CD23^lo^) and CD21/35^−^ CD23^−^ (B220^+^ CD21^lo^ CD23^lo^) B‐cell populations in 2‐ and 18‐month‐old mice. Median is shown, *n* = 5 or *n* = 6 mice per group. Results were analysed via Mann–Whitney *U*‐test. ***P < 0·001, **P < 0·01, *P < 0·05.

Next, FACS analysis was used to determine whether the changes to the microarchitecture of the MZ coincided with changes to the frequency and number of distinct splenic B‐cell populations. Cell suspensions were prepared from the same spleens as used for the histological analysis above. Significant decreases to immature B‐cell (at 12 and 15 months old) and CD3e^+^ T‐cell (at 15 months old) populations were observed. However, these were relative decreases due to accompanying increases in mature B cells at the same ages (Fig. 2b), and no consistent changes were observed throughout the observation period. Mature and follicular B‐cell populations, in contrast, were significantly increased at 12 and 15 months old, before decreasing in their representation at later ages (Fig. 2b). However, the total number of these B‐cell populations was unchanged in the spleens of 18‐month‐old mice (Fig. 2c). The frequency of MZ B cells was significantly increased in 15‐month‐old mice when compared with 2‐month‐old mice (Fig. 2b). When absolute numbers of MZ B cells were quantified in the spleens of 18‐month‐old mice, no significant changes were observed when compared with those from young spleens (*P* = 0·0519, Fig. 2c), although most aged mice had double the number of MZ B cells compared with young mice. These data contrast with those reported elsewhere, which suggested a decrease in MZ B cells in the spleens of 18‐month‐old BALB/c mice.^8^ Our data suggest that the effects of ageing on the abundance of MZ B cells may be background strain‐dependent. As anticipated, the frequency of age/autoimmune‐associated CD21/35^−^ CD23^−^ B cells was significantly increased at 15‐24 months of age (Fig. 2b), and their total numbers were significantly increased in 18‐month‐old mice when compared with young mice (Fig. 2c). These age/autoimmune‐associated B cells have been characterized elsewhere and will not be a primary focus of this study.^17^, ^18^, ^19^, ^20^ These data show that ageing is associated with significant dynamic changes to the frequency and number of certain B‐cell populations in the spleen.

### Ageing alters the shuttling and localization of MZ B cells

MZ B cells constantly shuttle between the follicle and MZ to collect blood‐borne complement‐opsonized antigens and deliver them to FDC in the B‐cell follicles.^3^, ^21^ At any time, typically 50% of the MZ B cells are in the MZ, the rest in the follicles.^3^ Whether the ageing‐related changes to the splenic MZ affected the follicular shuttling of MZ B cells was not known. To address this issue, an established antibody pulse‐labelling protocol was adopted to chronologically compare the frequency of these cells within the vascular compartment (MZ) and B‐cell follicles.^3^, ^22^ With this method a brief (5 min) intravenous exposure to PE‐conjugated anti‐CD21 monoclonal antibody (mAb) selectively labels the B cells exposed to peripheral blood (those within the MZ and bloodstream) by binding to their CD21. The CD21^+^ B cells within the follicle are protected from labelling at this time as the size of the PE prevents the mAb from accessing this site. By 20 min post‐injection with anti‐CD21‐PE mAb a greater proportion of the MZ B cells are labelled due to their migration between the follicles and MZ during the intervening period. Hence, by comparing the levels of PE^+^ cells at intervals after intravenous anti‐CD21‐PE mAb treatment, the kinetics of B‐cell localization and shuttling between the MZ and follicles can be determined.^3^, ^22^

**Figure 3:**
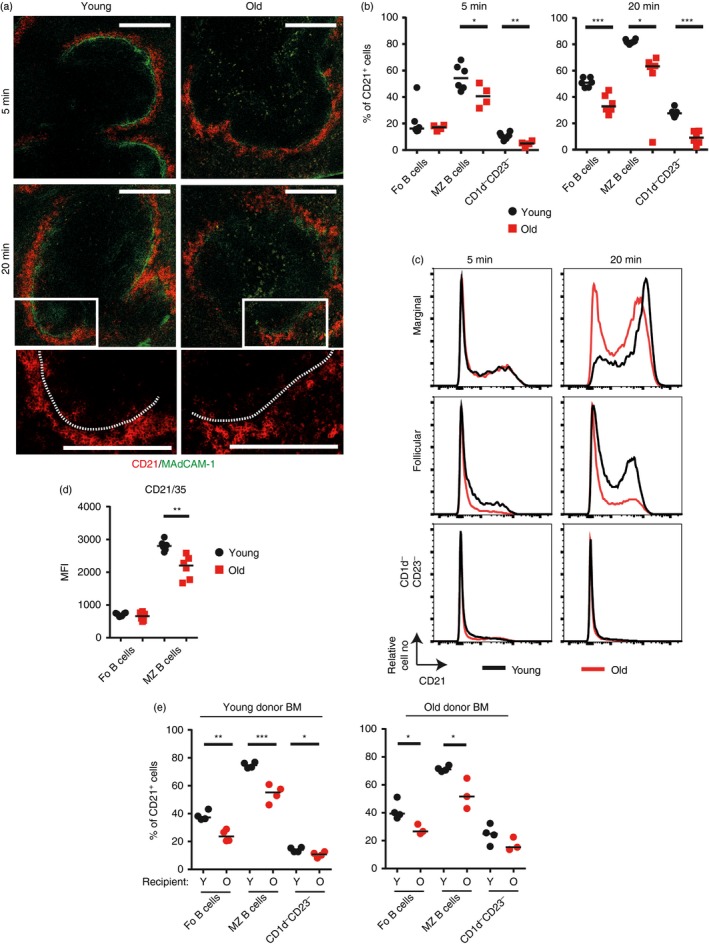
Decreased shuttling of B cells in the aged splenic environment. (a–c) Young and old mice were given 1 μg of anti‐CD21/35‐PE monoclonal antibody (mAb) intravenously then spleens collected for analysis 5 or 20 min later. Data are compiled from two experiments with a total of four to six mice per group. (a) Representative images of immunofluorescence histology on spleen sections from injected mice showing localization of CD21‐PE (red) and localization of MAdCAM‐1 (green). Enlargements show CD21‐PE movement into follicles at 20 min. Scale bar = 200 μm. (b) Percentage uptake of anti‐CD21/35‐PE by follicular (B220^+^ CD93^−^ CD23^+^ CD1d^−^), marginal zone (B220^+^ CD93^−^ CD23^−^ CD1d^hi^), or CD1d^−^ CD23^−^ (B220^+^ CD93^−^ CD23^−^ CD1d^−^) B cells is shown 5 or 20 min post‐injection in young (black circles) and old (red squares) mice. (c) Representative expression levels of CD21‐PE on follicular, marginal zone and CD1d^−^ CD23^−^ B cells in young (black line) and old (red line) mice. (d) Median fluorescence intensity of CD21/35 on follicular (B220^+^ CD93^−^ CD21^mid^ CD23^hi^) and marginal zone (B220^+^ CD93^−^ CD21^hi^ CD23^lo^) B cells in young (black circles) and old (red squares) mice. Data are compiled from two experiments with a total of four to six mice per group. (e) Lethally irradiated young or old recipient mice were transplanted with bone marrow from young or old donor mice. Ten weeks post‐reconstitution mice were given 1 μg of anti‐CD21/35‐PE monoclonal antibody (mAb) intravenously then spleens were collected for analysis 20 min later. Percentage uptake of anti‐CD21/35‐PE mAb by follicular (B220^+^ CD93^−^ CD23^+^ CD1d^−^), marginal zone (B220^+^ CD93^−^ CD23^−^ CD1d^hi^), or CD1d^−^ CD23^−^ (B220^+^ CD93^−^ CD23^−^ CD1d^−^) B cells is shown. Median is shown, *n* = 3 or *n* = 4 mice per group and is representative of two experiments for young donor bone marrow and one experiment for old donor bone marrow. Results were analysed using *t*‐test. ***P < 0·001, **P < 0·01, *P < 0·05.

Young (2 months) and old (18 months) mice were injected with anti‐CD21‐PE mAb and the distribution and localization of the anti‐CD21‐PE mAb‐bound B cells determined 5 and 20 min later. Immunohistochemical analysis confirmed the binding of anti‐CD21‐PE mAb by cells within the MZ in the spleens of mice from each age group at 5 min post‐injection (Fig. 3a). However, FACS analysis revealed that by 5 min after anti‐CD21‐PE mAb injection significantly fewer PE^+^ MZ B cells were in the MZ of spleens from old mice compared with young mice (Fig. 3b). This suggested a decreased localization of MZ B cells to the MZ and MZ sinus region in the spleens of old mice. Binding of anti‐CD21‐PE mAb by some follicular B cells was also evident at this time (Fig. 3b), consistent with the presence of recirculating follicular B cells in the MZ and blood.^3^, ^23^ By 20 min after anti‐CD21‐PE mAb injection the frequency of PE^+^ MZ B cells had increased to c. 80% in young mice (Fig. 3b), consistent with the shuttling of additional MZ B cells between the follicle and MZ during the intervening period.^3^ However, in the spleens of aged mice at 20 min after injection, the percentage of PE^+^ follicular, MZ and CD1d^−^ CD23^−^ B cells was significantly less than the young mice (Fig. 3b), indicating decreased movement of all subtypes of aged B cells between the MZ and follicle. These data clearly demonstrate that the localization and shuttling of B cells between the MZ and follicles is significantly impaired in the aged spleen. The amount of anti‐CD21‐PE mAb bound by aged MZ B cells was also reduced (Fig. 3c) and coincided with the significantly reduced expression of CD21 on their surfaces (Fig. 3d). This demonstrated that the ability of aged MZ B cells to acquire complement‐opsonized antigens via CD21 was also impaired.

To test whether the impaired B‐cell shuttling in aged spleens was due to the effects of ageing on leukocyte or stromal cell factors, young and old mice were *γ*‐irradiated and reconstituted with bone marrow from young or aged mice. Regardless of the age of the donor bone marrow the follicular shuttling of MZ B cells was significantly reduced in the spleens of old recipient mice, when compared with young recipients (Fig. 3e). These data clearly show that the effects of ageing on splenic stromal cells were predominantly responsible for the impaired follicular shuttling of MZ B cells in aged mice, rather than the ageing effects on the MZ B cells themselves.

### Altered migration towards CXCL13 and S1P by aged B cells

**Figure 4:**
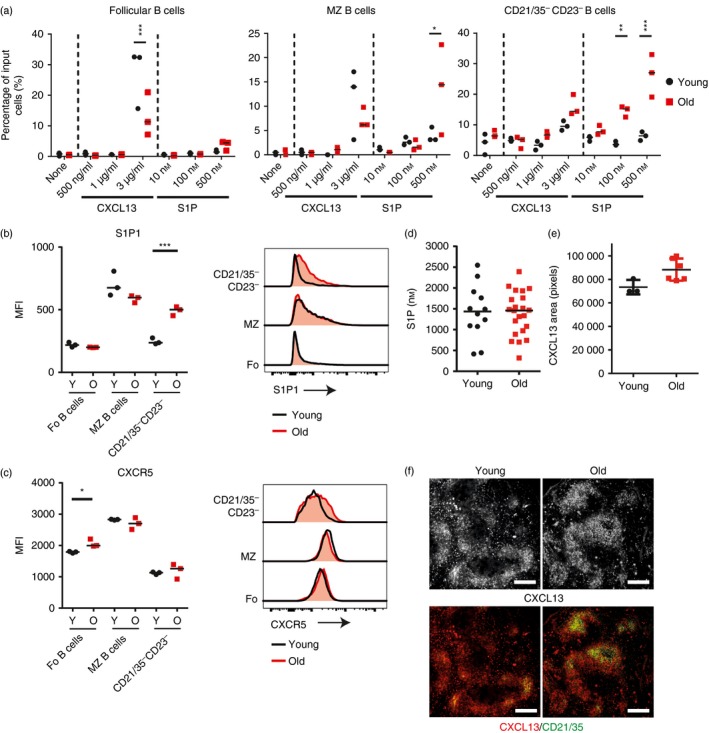
Altered chemotaxis of aged B cells to CXCL13 and sphingosine 1‐phosphate (S1P). (a) Chemotactic response of follicular (B220^+^ CD93^−^ CD21^mid^ CD23^hi^), marginal zone (B220^+^ CD93^−^ CD21^hi^ CD23^lo^) and CD21/35^−^ CD23^−^ (B220^+^ CD93^−^ CD21^lo^ CD23^lo^) B‐cell populations to indicated concentration of CXCL13 or S1P presented as the percentage of input of cells that have migrated through transwells into the lower chamber in a 4‐hr assay. Input cells were equal numbers of cells pooled from three young and three old mice and technical replicates are shown. Data are representative of two repeats and results were analysed via two‐way analysis of variance with Sidak's post‐test. Median fluorescence intensity of S1P_1_ (b) or CXCR5 (c) on follicular (B220^+^ CD93^−^ CD21^mid^ CD23^hi^), marginal zone (B220^+^ CD93^−^ CD21^hi^ CD23^lo^) and CD21/35^−^ CD23^−^ (B220^+^ CD93^−^ CD21^lo^ CD23^lo^) B‐cell populations from young and aged mice. Results were analysed using *t*‐test, *n* = 3 per group. (d) Concentration of S1P in the serum of young and aged mice, measured with HPLC. Results were analysed using Mann–Whitney *U‐*test, there was no significant difference. *n* = 12 young mice and 22 old mice. (e) CXCL13 area in the follicle of spleens from young and aged mice. Results were analysed via Mann–Whitney *U*‐test, there was no significant difference, *n* = 3 to *n* = 6 mice per group. (f) Representative histological staining of CXCL13 on young and aged spleens. Scale bars = 250 μm. ***P < 0·001, **P < 0·01, *P < 0·05.

CXCL13‐CXCR5 stimulation mediates the migration of B cells towards the follicles, whereas S1P_1_ and S1P_3_ mediate the reciprocal migration of B cells towards S1P in the marginal sinus of the MZ.^2^, ^3^ Whether ageing adversely affected the migration of MZ B cells towards these important chemoattractants was not known. Data from *in vitro* chemotaxis assays showed that the migration of aged follicular B cells towards CXCL13 was significantly reduced, whereas the migration of aged MZ B cells towards S1P was significantly increased (Fig. 4a). The expression of S1P_1_ on young and aged follicular or MZ B cells was similar (Fig. 4b), although a small but significant increase in CXCR5 expression was observed on aged follicular B cells (Fig. 4c). Aged CD21/35^−^ CD23^−^ B cells also displayed a significant increase in their ability to migrate towards S1P (Fig. 4a), and expressed increased levels of S1P_1_ on their surfaces (Fig. 4b). Unfortunately, no suitable mAb were available to allow detection of S1P_3_ by FACS. S1P concentrations in the sera of young and aged mice were measured via HPLC and no significant difference was observed (Fig. 4d). It is plausible that the increased migration of aged MZ B cells towards S1P, and decreased migration of aged follicular B cells towards CXCL13, may contribute to their enhanced retention within the MZ. In contrast to the above effects, the migration of follicular or MZ B cells towards 2‐arachidonylglycerol, a ligand also important in MZ B‐cell migration and retention,^22^, ^24^ was equivalent in cells from young and aged mice (data not shown).

Immunohistochemical analysis showed that in the spleens of young mice CXCL13 was typically centralized around FDC with additional punctate immunostaining within the red pulp (Fig. 4f). Although there was no significant difference in the area of follicular CXCL13 expression between young and age mice (Fig. 4e), in the spleens of aged mice abundant CXCL13 expression was detected in the B‐cell follicles but it was not localized to the FDC, and also appeared to be reduced in the white pulp (Fig. 4f). These results have a similar trend to those previously published in BALB/c mice,^9^ indicating similarities between the mouse species in this regard.

### Decreased uptake of type 2 TI antigen with age

**Figure 5:**
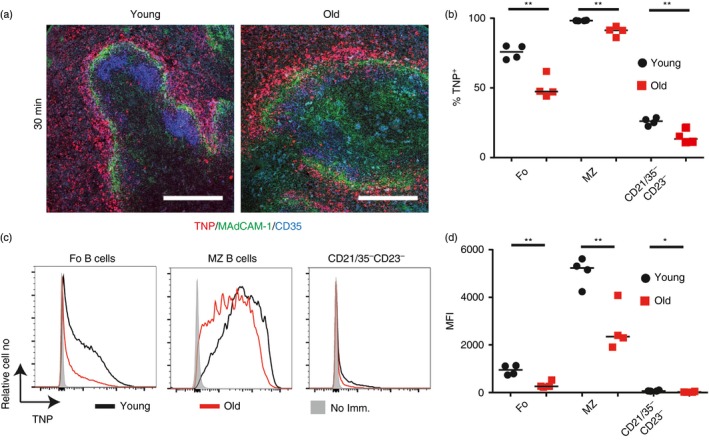
B cells in aged mice have a decreased uptake of trinitrophenyl (TNP) ‐Ficoll. Young and old mice were immunized with TNP‐Ficoll. After 30 min spleens were collected for analysis. (a) Representative immunofluorescence images of TNP localization in the spleens of young and old mice (TNP – red, MAdCAM‐1 – green, CD35 – blue). Scale bars = 200 μm. (b) A flow cytometric analysis was performed to determine the percentage of follicular (B220^+^ CD21^mid^ CD23^hi^), marginal zone (B220^+^ CD21^hi^ CD23^lo^) and CD21/35^−^ CD23^−^ (B220^+^ CD21^lo^ CD23^lo^) B cells that are binding TNP‐Ficoll. (c) Expression levels of TNP on the follicular (B220^+^ CD21^mid^ CD23^hi^), marginal zone (B220^+^ CD21^hi^ CD23^lo^) and CD21/35^−^ CD23^−^ (B220^+^ CD21^lo^ CD23^lo^) B cells in non‐immunized, young and old mice as determined via flow cytometry. (d) Median fluorescence intensity levels of TNP on follicular, marginal zone and CD21/35^−^ CD23^−^ B cells. Results are representative of two experiments. Median is shown, *n* = 4 mice per group, and analysis was via *t*‐test. **P < 0·01, *P < 0·05.

The MZ B cells play important roles in the rapid induction of TI responses to blood‐borne polysaccharide antigen,^22^, ^25^, ^26^ but whether this ability was affected by ageing was not known. Therefore we first determined whether ageing affected the ability of MZ B cells to acquire TNP‐Ficoll, a TI‐type 2 model antigen.^27^ Young and aged mice were injected intravenously with TNP‐Ficoll and spleens were collected 30 min later. Immunohistochemical analysis demonstrated TNP localization to the MZ region young and aged spleens (Fig. 5a). However, FACS analysis showed that the percentage of MZ, follicular and CD21/35^−^ CD23^−^ B cells that had acquired TNP‐Ficoll was significantly reduced in aged spleens (Fig. 5b). The amount of TNP‐Ficoll bound to the surfaces of these aged B‐cell populations was also significantly reduced when compared with B cells from young mice (Fig. 5c, d). These results clearly demonstrate that aged MZ B cells have a significantly decreased ability to acquire TI‐type 2 antigens.

### Effects of ageing on the response to TI antigen

**Figure 6:**
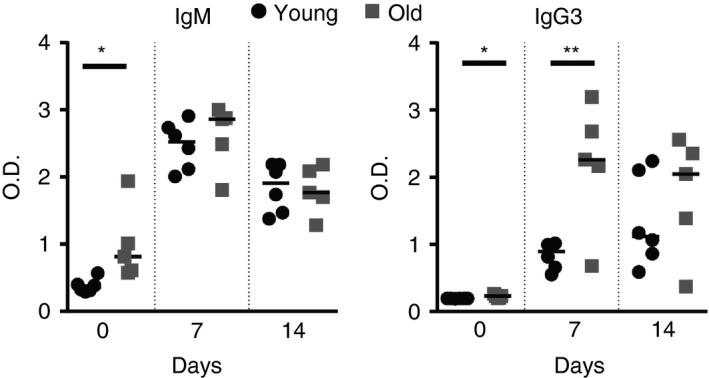
Decreased long‐term response of aged mice to trinitrophenyl (TNP) ‐Ficoll. Mice were given TNP‐Ficoll and serum was collected from the tail vein before immunization and 7 or 14 days post‐immunization. ELISAs were performed to measure TNP‐specific IgM or IgG3 levels. Graphs show median OD_405_; *n* = 5 or *n* = 6 mice per group. Results were analysed using one‐way analysis of variance with Tukeys post‐test. **P < 0·01, *P < 0·05.

Next, young and aged mice were immunized with the model TI type 2 antigen TNP‐Ficoll and serum levels of IgM and IgG3 were measured 7 or 14 days later. The basal level of TNP‐specific IgM and IgG3 was significantly higher in the sera of aged mice (Fig. 6). Post‐immunization, the levels of TNP‐specific IgM produced were similar in mice from each age group (Fig. 6). Conversely, the levels of TNP‐specific IgG3 were significantly higher in the sera of aged mice 7 days after immunization (Fig. 6). These data show that despite the reduced uptake and follicular‐shuttling of TNP‐Ficoll by aged MZ B cells (Fig. 5), their ability to mount an antigen‐specific IgM and IgG3 response following immunization with this TI‐type 2 antigen was not adversely affected.

**Figure 7:**
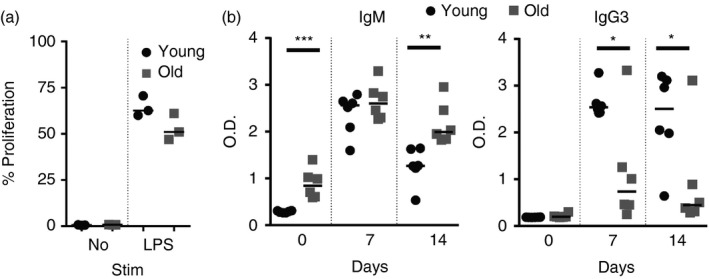
Aged B cells have a decreased response to trinitrophenyl–lipopolysaccharide (TNP‐LPS). (a) B cells were enriched from the spleens of young or old mice, labelled with Cell Trace Far Red and cultured *in vitro* in media alone or containing 10 μg/ml LPS for 48 hr. The percentage of B cells that proliferated is shown. (b) Mice were given TNP‐LPS. Serum was collected from the tail vein before immunization and 7 or 14 days post‐immunization. ELISAs were performed to measure TNP‐specific IgM or IgG3 levels. Graphs shown median OD_405_. *n* = 6 mice per group. Results were analysed by one‐way analysis of variance with Tukeys post‐test. ***P < 0·001, **P < 0·01, *P < 0·05.

We also determined whether ageing affected the induction of an immune response to the model TI‐type 1 antigen, TNP‐LPS.^28^ B cells were enriched from young and aged mice, stimulated *in vitro* with LPS and 48 hr later cell proliferation was compared by FACS. MZ B cells vigorously proliferate in response to LPS stimulation.^26^, ^29^ Our data show that this ability was not affected by ageing as the proliferation of LPS‐stimulated B cells from young and aged mice was similar (Fig. 7a). Unfortunately, no suitable antibodies currently exist to assess the capture of LPS by B cells; however, MZ B cells from young and aged mice expressed equivalent levels of TLR4 levels on their surface (data not shown).

Marginal zone B cells also produce high levels of IgM and IgG3 in response to LPS stimulation.^26^, ^28^, ^29^ Therefore, young and aged mice were next immunized with TNP‐LPS and serum levels of IgM and IgG3 were compared. As observed above, the basal level of TNP‐specific IgM was significantly higher in the sera of aged mice (Fig. 7b). Post‐immunization, the level of TNP‐specific IgM was similar in sera from each age group at 7 days after immunization, but was significantly higher in aged mice by 14 days (Fig. 7b). In contrast, the levels of TNP‐specific IgG3 was significantly reduced in the sera of aged mice at both 7 and 14 days after immunization (Fig. 7b). These data show that antigen‐specific IgG3 responses to the model TI‐type 1 antigen TNP‐LPS were significantly impaired in aged mice.

## Discussion

The splenic MZ is a specialized microenvironment that plays an important role in the rapid capture and clearance of blood‐borne pathogens/antigens. Significant disturbances to the microarchitecture of the MZ have been described, but little was known of when these changes were first encountered. In the current study we provide a detailed chronological account of the changes to the microarchitecture of the splenic MZ at 3‐monthly intervals throughout the life course. We show that significant disturbances to the MAdCAM‐1^+^ sinus lining cells within the MZ were evident from as early as 9 months old and preceded the changes to the positioning of the MZ B cells and MZ metallophilic macrophages. MZ B cells are strategically positioned to capture blood‐borne antigen and immune complexes and deliver them to FDC in the follicles. We show that in the spleens of aged mice, antigen capture and follicular shuttling by MZ B cells was adversely affected. Whereas aged follicular B cells had decreased migration to the follicular chemoattractant CXCL13, the ability of aged MZ B cells to migrate towards the MZ chemoattractant S1P was increased. This suggested that aged MZ B cells had a greater propensity to be retained within the MZ. The extrinsic impairment in aged B‐cell migration towards the MZ was confirmed by the use of bone marrow chimeras. The follicular shuttling of MZ B cells derived from either young or aged bone marrow was similarly reduced in aged recipient spleens, demonstrating that ageing effects on splenic stromal cells were responsible for the impaired follicular shuttling of MZ B cells. MZ B cells mount rapid antigen‐specific IgM and IgG3 responses to TI antigen. Our data show that aged MZ B cells have a significantly decreased ability to acquire certain TI antigens. Furthermore, the ability of aged mice to produce immunoglobulins in response to the TI‐type 1 antigen TNP‐LPS was also impaired. MZ B cells are important first responders to blood‐borne pathogens. A thorough analysis of the cellular and molecular mechanisms that underpin the ageing‐related decline in the status of the MZ and MZ B cells will aid our understanding of the factors that influence susceptibility to blood‐borne pathogens and their antigens, and improve immunity in the elderly.

B cells within the MZ are continually exposed to S1P in the blood.^2^ MZ B cells express the S1P receptors S1P_1_ and S1P_3_ highly, facilitating their S1P‐mediated attraction to the MZ.^2^, ^3^, ^25^ Continual S1P stimulation is required to retain MZ B cells in the MZ and it is the desensitization of S1P receptors on MZ B cells that enables them to overcome their attraction towards S1P in the blood^30^ and migrate into the follicles. Stromal cells, including FDC, within the B‐cell follicles express CXCL13,^31^ which stimulates the attraction of CXCR5‐expressing B cells towards the follicles.^2^, ^3^, ^25^, ^32^ Once within the follicles, the cells up‐regulate their expression of S1P_1_ and S1P_3_ and are attracted in an S1P‐dependent manner back to the MZ. In the current study we revealed significant changes in the migration of aged B cells to these chemoattractants. Follicular B cells had increased CXCR5 expression but decreased migration towards CXCL13. Implying that aged B cells may have impaired downstream functioning from CXCR5, as one would anticipate increased CXCR5 expression to enhance migration towards CXCL13.^33^ Aged MZ B cells displayed increased migration to S1P, but the expression levels of S1P_1_ and S1P in the sera were similar in young and old mice. Whether S1P_3_ is altered on aged MZ B cells is uncertain, as no suitable FACS antibody currently exists. However, these data show that aged MZ B cells are not intrinsically impaired in their ability to migrate towards the MZ, suggesting that the ageing‐related structural changes to the MZ have a major impact on the migration of aged B cells to and within it. The disordered and expanded MAdCAM‐1^+^ stromal cell layer in the spleens of aged mice may physically inhibit the shuttling of MZ B cells between the MZ and follicle as well as impede the efficient percolation of blood through it. The increased migration of MZ B cells towards the S1P may also contribute towards their enhanced retention within the MZ.

The disturbances to the MAdCAM‐1^+^ stromal cell layer in aged spleens preceded disturbances to the distribution of MZ B cells and the CD169^+^ MZ metallophilic macrophages. This implies that the effects of ageing on the MAdCAM‐1^+^ stromal cell layer were, at least in part, responsible for the effects observed on the distribution of MZ B cells and CD169^+^ macrophages within this region. The distribution of MAdCAM‐1^+^ stromal cells and CD169^+^ MZ metallophilic macrophages is disturbed in the spleens of S1P_3_^−/−^ mice, indicating an important role for S1P–S1P_3_ signalling in the organization of the marginal sinus.^25^ Whether the effects of ageing on the MZ microarchitecture were similarly a consequence of reduced S1P_3_ receptor expression by splenic stromal cells remains to be determined. However, our data clearly show that these effects on the MZ observed in aged mice were not to the result of reduced levels of S1P within the bloodstream.

The MZ B cells capture complement‐opsonized antigen or immune complexes via the complement receptors CR2/CR1 (CD21/35)^28^ and transfer them to FDC upon migration into the follicle.^3^, ^4^, ^21^ The expression of CR2/CR1 on aged MZ B cells was decreased and coincided with their decreased uptake of TI‐type 2 antigen. A role for the engagement of MARCO^+^ MZ macrophages in the regulation of antigehn uptake and transport by MZ B cells through CR2/CR1 has been proposed.^34^ However, the distribution of MZ macrophages, and their expression of MARCO, was not adversely affected in aged spleens, suggesting that the reduced expression of CR2/CR1 on aged MZ B cells was unlikely to be due to ageing effects on MZ macrophages.

The MZ B cells rapidly mount TI IgM and IgG3 responses to lipid and capsular carbohydrate antigen.^35^, ^36^, ^37^ We showed that this ability was significantly altered in aged mice. Despite the reduced uptake of the model TI‐type 2 antigen TNP‐Ficoll by aged MZ B cells, aged mice produced significantly higher levels of IgG3 within 7 days of immunization compared with young mice. Increased IgG3 production by aged mice has also been observed in response to *S. pneumoniae* vaccination.^38^ These characteristics contrasted with those observed following immunization with TNP‐LPS, a TI‐type 1 antigen. As MZ B cells can undergo class switch recombination independently of germinal centres,^39^ external factors from the localization and shuttling of MZ B cells are contributing to the altered IgM and IgG3 production by aged MZ B cells. B‐cell responses to certain TI‐type 1 antigens are mediated through the co‐activation of B cells, e.g. TLR4, the receptor for LPS. MZ B cells proliferate and rapidly secrete high levels of IgM and IgG3 in response to LPS stimulation.^26^, ^28^, ^29^ However, cell proliferation in response to LPS stimulation was not affected in aged mice, nor was the level of TLR4 expression. However, aged mice produced less IgG3 following immunization with TNP‐LPS than young mice, indicative of impaired signalling pathways in response to TNP‐LPS. Conversely, the increased IgG3 production in response to TNP‐Ficoll may be indicative of a lack of negative feedback in this signalling pathway. These data suggest that in aged individuals the ability to regulate responses to TI antigen may be impaired. Furthermore, the decreased shuttling seen by both follicular and MZ B cells inhibits antigen delivery to the follicle and FDC, and may also contribute towards the impaired T‐cell‐dependent immune responses and germinal centre formation observed with age.^40^, ^41^, ^42^, ^43^

Data in the current study show how ageing can have a significant impact on the migration and function of MZ B cells. Invasive pneumococcal disease from *S. pneumoniae* infection is a leading cause of mortality in individuals > 65 years old,^14^ and vaccine efficacy against this disease is decreased in the elderly.^15^ MZ B‐cell deficiencies are associated with elevated risk of pneumococcal disease and poor antibody responses to microbial capsular polysaccharides,^12^, ^13^ suggesting that effects of ageing on the MZ may similarly impact on the ability of MZ B cells in the elderly to acquire antigen to mount effective TI‐antibody responses. *Vibrio cholerae* LPS is considered an important protective antigen for a cholera subunit vaccine. As MZ B cells play an important role in the induction of anti‐*V. cholerae* LPS immunoglobulin responses^44^ our data suggest that such vaccines may also be less effective in the elderly. In the spleens of aged mice antigen capture and follicular shuttling by MZ B cells was impaired. Our data suggested that ageing effects on splenic stromal cells were predominantly responsible for the impaired follicular shuttling of MZ B cells. However, the precise mechanisms responsible for the ageing‐related disturbances to the stromal compartment remain to be determined. A thorough analysis of the molecular mechanisms that mediate the ageing‐related disturbances to the microarchitecture of the MZ and MZ B‐cell function will aid our understanding of the factors that influence susceptibility to certain blood‐borne pathogens and help to identify novel approaches to improve vaccine efficacy in the elderly.

## Author contributions

V.M.T. performed the experiments. V.M.T. and N.A.M. designed the experiments, interpreted the data and wrote the manuscript.

## Disclosures

The authors report no conflicts of interest.

## Supporting information


**Figure S1.** Quantification methods to assess splenic disruption via immunofluorescence
