# Supplementary figures and images for: Ageing adversely affects the migration and function of marginal zone B cells
Source: Immunology. 2017 May 4;151(3):349–62. doi: 10.1111/imm.12737 (PMC5461100; doi:10.1111/imm.12737)

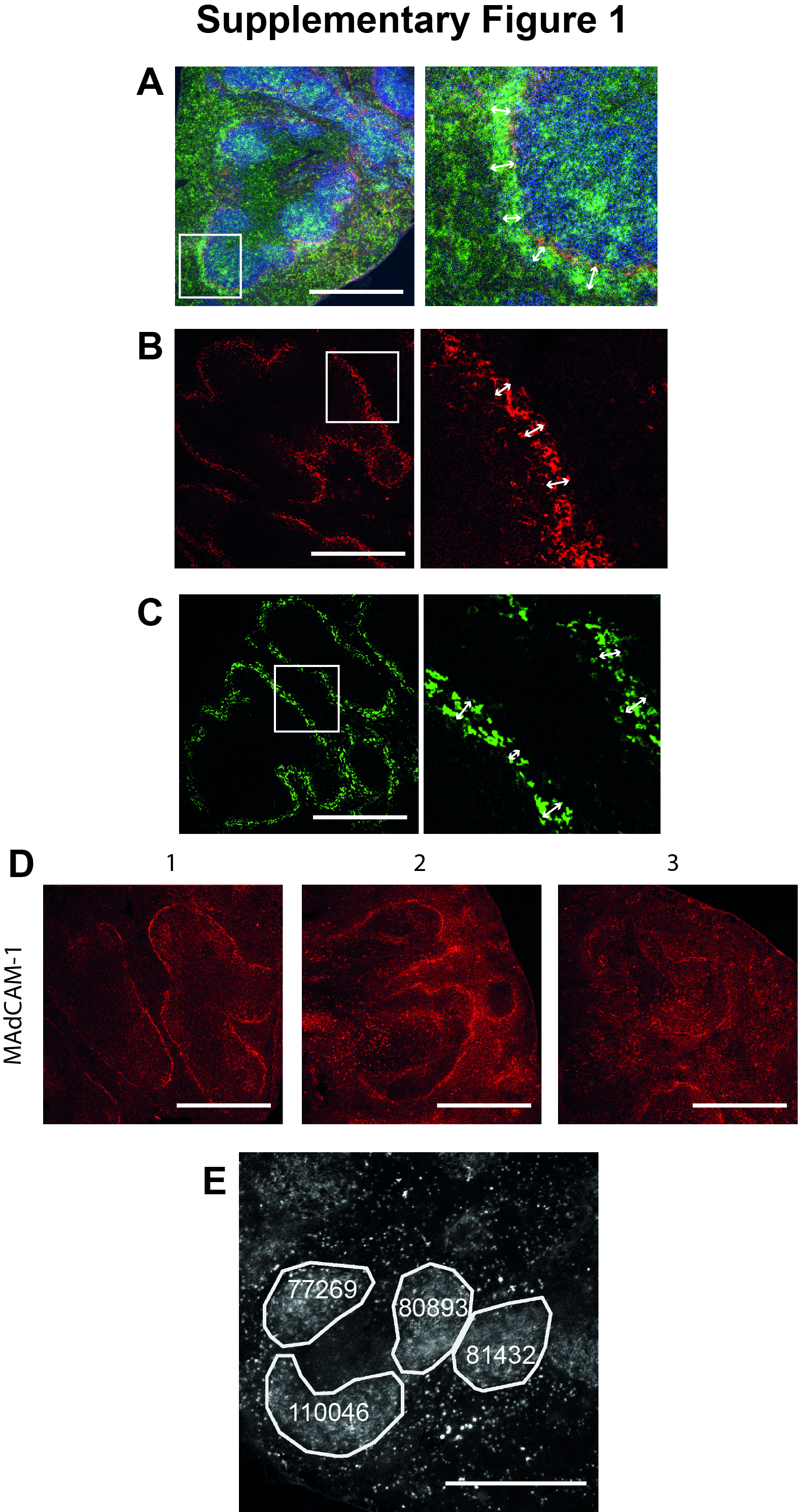

Supplement: Supplementary file 1 — Figure S1. Quantification methods to assess splenic disruption via immunofluorescence [file IMM-151-349-s001.tif]
